# The Metrical System in Great Britain

**Published:** 1879-04

**Authors:** 


THE METRICAL SYSTEM IN GREAT BRITAIN. — It is a curious fact that the metrical system, which has been permissive in Great Britain, ceased to be so on the 1st of January, 1879, when the new act relating to weights and measures went into force, at which date it became lawful to buy and sell only by imperial measures, among which those of the metrical system are not included.—*Drug. Cir. and Chem. Gaz.*

---

THE man who gives as his excuse for not subscribing to a new journal, "I haven't time to read those I already take," is, in nine cases out of ten, a poor tool. The busiest, most successful men in the practice of medicine, are those who read most and write most. They are the systematic workers; it is only the dawdler and the drone who "can't find time to read."—*Maryland Med. Jour.*

